# Indoxyl sulfate in the gut–kidney axis: from diet-microbiome interactions to renal injury and targeted therapies

**DOI:** 10.3389/fnut.2026.1881019

**Published:** 2026-09-16

**Authors:** Xiangning Huang, Xinyu Yang, Jiawang Huang, Wang Tao, Yunfeng Yu, Rong Yu

**Affiliations:** 1 School of Traditional Chinese Medicine, Hunan University of Chinese Medicine, Changsha, China; 2 Department of Endocrine, The First Hospital of Hunan University of Chinese Medicine, Changsha, China

**Keywords:** chronic kidney disease, indoxyl sulfate, mechanisms of renal injury, protein-bound uremic toxin, targeted therapy

## Abstract

Indoxyl sulfate (IS) is a prototypical protein-bound uremic toxin, linking dietary tryptophan metabolism by gut microbiota to renal injury. Derived from dietary tryptophan metabolized by gut microbiota into indole, which is subsequently transported to the liver and sulfated to form IS. In circulation, IS binds serum albumin and is actively secreted into urine via renal tubular organic anion transporters. In chronic kidney disease (CKD), impaired renal clearance and altered albumin-binding capacity contribute to IS accumulation and an increased free fraction. IS exerts nephrotoxic effects through multiple mechanisms. As an endogenous ligand of the aryl hydrocarbon receptor (AHR), it activates AHR signaling and contributes to mitochondrial dysfunction, oxidative stress, inflammation, regulated cell death, and renal fibrosis through interconnected downstream pathways. Therapeutic strategies targeting IS include dietary and microbiota-based interventions, oral adsorbent AST-120, modified dialysis approaches, and traditional Chinese medicine interventions. Although these strategies may reduce IS exposure or its downstream effects, the current evidence is predominantly derived from preclinical studies and surrogate-endpoint assessment. Further well-designed clinical trials are needed to determine whether reducing IS exposure can translate into sustained improvements in renal and patient-centered outcomes.

## Graphical abstract

**Figure:**
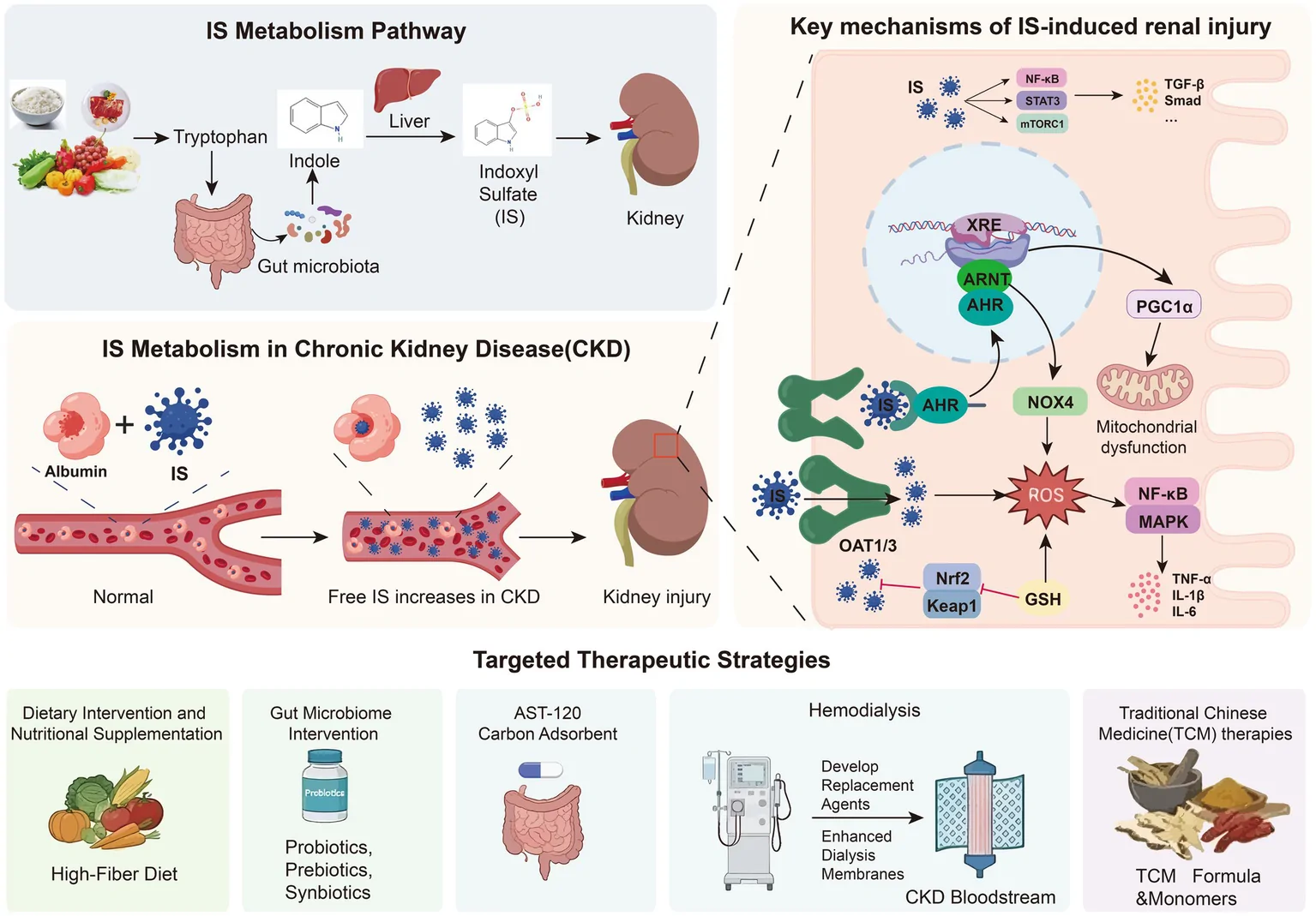
Dietary tryptophan is metabolized by gut microbiota to indole, which is subsequently converted to IS in the liver. In circulation, IS binds serum albumin and is secreted through renal tubular OAT1/3. In CKD, impaired renal clearance and an increased free fraction promote IS accumulation. IS activates AHR and multiple interconnected downstream pathways, including NADPH oxidase/NOX4/ROS, Nrf2/Keap1, NF-κB/MAPK, STAT3, and mTORC1 signaling, contributing to mitochondrial dysfunction, oxidative stress, inflammation, regulated cell death, and renal fibrosis. Current IS-targeted strategies include dietary and nutritional interventions, gut microbiota modulation, AST-120, modified dialysis approaches, and TCM. However, the clinical efficacy and long-term benefits of these interventions remain to be established. AHR, aryl hydrocarbon receptor; ARNT, aryl hydrocarbon receptor nuclear translocator; AST-120, an oral carbon adsorbent; CKD, chronic kidney disease; GSH, glutathione; IL-1β, interleukin-1β; IL-6, interleukin-6; IS, indoxyl sulfate; Keap1, Kelch-like ECH-associated protein 1; MAPK, mitogen-activated protein kinase; mTORC1, mechanistic target of rapamycin complex 1; NF-κB, nuclear factor kappa B; NOX4, NADPH oxidase 4; Nrf2, nuclear factor erythroid 2–related factor 2; OAT1/3, organic anion transporter 1/3; PGC1α, peroxisome proliferator-activated receptor γ coactivator 1α; ROS, reactive oxygen species; Smad, Sma and Mad-related protein; STAT3, signal transducer and activator of transcription 3; TCM, Traditional Chinese medicine; TGF-β, transforming growth factor-β; TNF-α, tumor necrosis factor-α; XRE, xenobiotic response element.

## 1 Introduction

Chronic kidney disease (CKD) is a clinical syndrome characterized by persistent structural and/or functional abnormalities of the kidneys (1). CKD is diagnosed based on evidence of kidney damage or reduced glomerular filtration rate (GFR) persisting for at least 3 months (1). Common etiologies of CKD include diabetes mellitus, hypertension, and glomerulonephritis (2). Recent epidemiological data indicate that approximately 850 million individuals worldwide are living with CKD (2). Among these patients, approximately 4 million have progressed to end-stage renal disease (ESRD) (2). Projections indicate that by 2050, CKD may become the fifth leading global cause of death (2). This growing disease burden is expected to impose substantial economic and social pressures on global healthcare systems and public health infrastructures. Therefore, a comprehensive understanding of CKD pathogenesis, particularly with respect to early diagnosis and therapeutic intervention, has become a critical priority in contemporary clinical practice and translational research.

As renal function progressively declines in CKD patients, metabolites normally excreted by the kidneys progressively accumulate in the body (3). When these metabolites induce organ senescence and dysfunction, they are termed uremic toxins (4–6). Consequently, the resulting systemic organ dysfunction is referred to as uremic syndrome (4, 5). According to their physicochemical properties and dialytic removability, uremic toxins are generally classified into three categories: small water-soluble compounds, protein-bound compounds, and middle molecules (7). Among these, protein-bound uremic toxins (PBUTs) exist as high-molecular-weight complexes in the bloodstream due to their strong affinity for human serum albumin (HSA) (5). PBUTs are difficult to remove using conventional dialysis techniques and are therefore considered major contributors to inadequate toxin clearance during dialysis (5). Indoxyl sulfate (IS), a prototypical member of the PBUT family, occupies a pivotal position in uremic toxin research. IS was first isolated and identified in 1911 and has been reported to accumulate at significantly elevated concentrations in the blood of CKD patients compared with healthy individuals (8). Beyond its effects on the kidney, IS has been implicated in injury across multiple organ systems, including the cardiovascular, nervous, and skeletal systems. Elevated IS levels have been associated with arrhythmias (9), cognitive dysfunction (10), obstructive sleep apnea (11), and osteoporosis (12). These findings highlight the systemic toxicity of IS and its potential contribution to the multisystem complications of CKD. However, the kidney remains a major target of IS toxicity, where IS can directly induce tubular cell death, promote mesangial cell proliferation, and stimulate renal fibrosis, thereby exacerbating the progression of kidney disease (13).

Although previous reviews have extensively summarized the pathophysiological mechanisms and therapeutic potential of IS, most have focused on its toxicity, downstream signaling pathways, and associations with various disease complications (8, 14–16). However, the upstream determinants of IS production, particularly the interactions among dietary tryptophan, gut microbial metabolism, and IS accumulation, have received comparatively less integrated attention. Therefore, this review takes a nutrition–gut microbiota–IS–kidney axis perspective, integrating the nutritional and microbial determinants of IS production with its downstream renal injury mechanisms and emerging IS-targeted therapeutic strategies. Particular emphasis is placed on microbiota-based and nutritional approaches to modulate IS metabolism, while critically considering the strength of available evidence and their translational potential for CKD management.

## 2 Metabolism and biological characteristics of IS

### 2.1 Metabolism and renal elimination of IS: from dietary tryptophan to urinary excretion IS

Dietary tryptophan is the primary precursor of IS and represents a key metabolic link between dietary intake, gut microbial activity, and systemic exposure to uremic toxins. Following its delivery to the colon, dietary tryptophan is metabolized by tryptophanase-producing gut microbiota to generate indole, which serves as a key microbial precursor for the subsequent hepatic biosynthesis of IS. Representative taxa involved in this process include *Bacteroides thetaiotaomicron*, *Bacteroides salyersiae*, *Prevotella* sp., CAG:5226, and Selenomonadales (17, 18). Following intestinal production and absorption, indole enters the liver via the systemic circulation, where it is hydroxylated into 3-hydroxyindole (indoxyl) by the hepatic microsomal cytochrome P450 enzyme CYP2E1 (19). Thereafter, 3-hydroxyindole undergoes sulfation catalyzed by sulfotransferase family 1A member 1 (SULT1A1), ultimately generating IS (20). Notably, although colonic epithelial cells (HT-29 Glc^−/+^ cells) also express CYP2E1 and are capable of converting indole into IS, their contribution to this metabolic process appears to be relatively limited (21).

Upon formation, IS, as a highly protein-bound uremic toxin, cannot freely cross the glomerular filtration barrier (22, 23). The majority of circulating IS is bound to albumin, primarily HSA (22, 23). In the kidney, organic anion transporters OAT1/SLC22A6 and OAT3/SLC22A8 primarily mediate the basolateral uptake of IS into renal tubular cells, whereas ATP-binding cassette transporter subfamily G member 2 (ABCG2) contributes to its apical efflux into the tubular lumen (22, 23). The kidney therefore plays a major role in the elimination of IS through tubular secretion (22, 23). Under conditions of renal insufficiency, compensatory enhancement of IS excretion through the gastrointestinal tract may occur to minimize its systemic accumulation (24).

### 2.2 Protein binding and IS accumulation in CKD

As a representative member of PBUTs, IS is characterized by its strong binding affinity for HSA. HSA is the most abundant plasma protein and provides the principal binding site for circulating IS (25, 26). IS predominantly binds to Sudlow site II of HSA, a high-affinity hydrophobic ligand-binding pocket (27–30). The binding constant ranges from 9.1 to 16.1 × 10^5^/M, corresponding to a dissociation constant (*K*D) of 0.6–1.1 μM (27–29). This high-affinity interaction substantially limits the glomerular filtration and conventional dialysis clearance of IS, thereby favoring its persistence in the circulation (27, 31–33).

Clinical studies indicate that high-affinity PBUTs such as IS accumulation in CKD is primarily associated with impaired renal clearance and alterations in HSA-binding capacity, which increase the circulating free fraction of IS and may enhance its biological toxicity (34). Multiple cohort studies have demonstrated a strong correlation between IS accumulation and estimated glomerular filtration rate (eGFR) (*R*^2^ = 0.55, *p* < 0.001) (35). Serum IS concentrations progressively increase with declining eGFR (35–37). In patients with mild CKD, serum IS concentrations are approximately 2–3-fold higher than those in healthy individuals (37, 38). In severe CKD, serum IS concentrations may reach 43–90-fold higher than those observed in healthy populations (39).

In summary, dietary tryptophan metabolism by the gut microbiota represents the initial step in the metabolic pathway leading to IS formation, with indole serving as a key microbial precursor for subsequent hepatic IS biosynthesis. Once formed, IS is predominantly eliminated through renal tubular secretion, whereas its high-affinity binding to serum albumin limits glomerular filtration and conventional dialysis clearance. In CKD, impaired renal clearance and altered albumin-binding capacity contribute to increased circulating free IS and substantial systemic accumulation. This metabolic and clearance profile provides a mechanistic basis for the persistent exposure to IS observed in CKD and may contribute to its renal and systemic toxicity. Although limited studies have reported that IS may exert anti-breast and anti-colorectal cancer effects under specific conditions (40, 41), its nephrotoxic effects remain the primary focus of research on its biological toxicity.

## 3 From gut microbial metabolism to IS-induced renal injury

### 3.1 AHR signaling as an upstream regulatory hub

As a potent endogenous AHR ligand, IS exhibits approximately 500-fold greater transcriptional activation potency in human cells than in mouse cell lines (42). Under normal physiological conditions, AHR functions as a critical transcription factor involved in maintaining normal kidney development and function (43, 44). In its inactive state, AHR resides in the cytoplasm, where it forms a stable multiprotein complex with heat shock protein 90 (Hsp90) and other chaperone proteins (43, 44). Upon binding of exogenous or endogenous ligands, including IS, AHR translocates from the cytoplasm to the nucleus (43, 44). Subsequently, AHR associates with the AHR nuclear translocator (ARNT) to form a heterodimer (43, 44). The resulting heterodimer subsequently recognizes and binds to xenobiotic response elements (XREs) located within the promoter regions of target genes, such as *CYP1A1* and *CYP1B1* (43, 45, 46). This binding event initiates transcriptional activation and subsequently upregulates the expression of downstream target genes (43, 45, 46). Finally, activated AHR is recognized and degraded through the ubiquitin–proteasome system, thereby completing the negative-feedback regulation of the signaling pathway (47).

Clinical and observational evidence supports an association between IS exposure, AHR activity, and renal dysfunction. In patients with CKD, serum AHR-activating potential (AHR-AP) increases with declining renal function and correlates positively with circulating IS levels (48). Consistent with these findings, higher AHR transcriptional activity in patients with diabetic kidney disease (DKD) is associated with increased urinary albumin and serum creatinine levels (49). However, these clinical observations remain associative and cannot establish whether aberrant AHR activation directly contributes to CKD progression. Experimental studies provide further mechanistic support for the involvement of IS-mediated AHR activation in renal injury. Specifically, elevated serum IS levels promoted aberrant AHR activation in mouse models of adenine-induced nephropathy and ischemia/reperfusion injury (50). Together with intervention studies targeting AHR, these findings provide stronger experimental support for a pathogenic role of AHR, although the extent to which this mechanism operates in human CKD remains to be established.

Importantly, studies in different renal cell types have identified distinct downstream effects of AHR activation. In tubular epithelial cells, aberrant AHR activation enhances the ubiquitination and degradation of peroxisome proliferator-activated receptor γ coactivator-1α (PGC1α), thereby impairing mitochondrial biogenesis and mitochondrial function and ultimately promoting tubular epithelial cell senescence and fibrosis (51). In mesangial cells, AHR activation promotes mesangial cell activation and macrophage infiltration, contributing to extracellular matrix accumulation (52). In TCMK-1 renal tubular epithelial cells, AHR activation increases the expression of the methyltransferase EZH2, which further promotes cellular senescence (53), as illustrated in Figure 1. Conversely, AHR silencing or knockdown, as well as pharmacological inhibition of AHR using CH-223191, effectively alleviates these pathogenic effects and suppresses IS-mediated renal injury (51–54).

**Figure 1:**
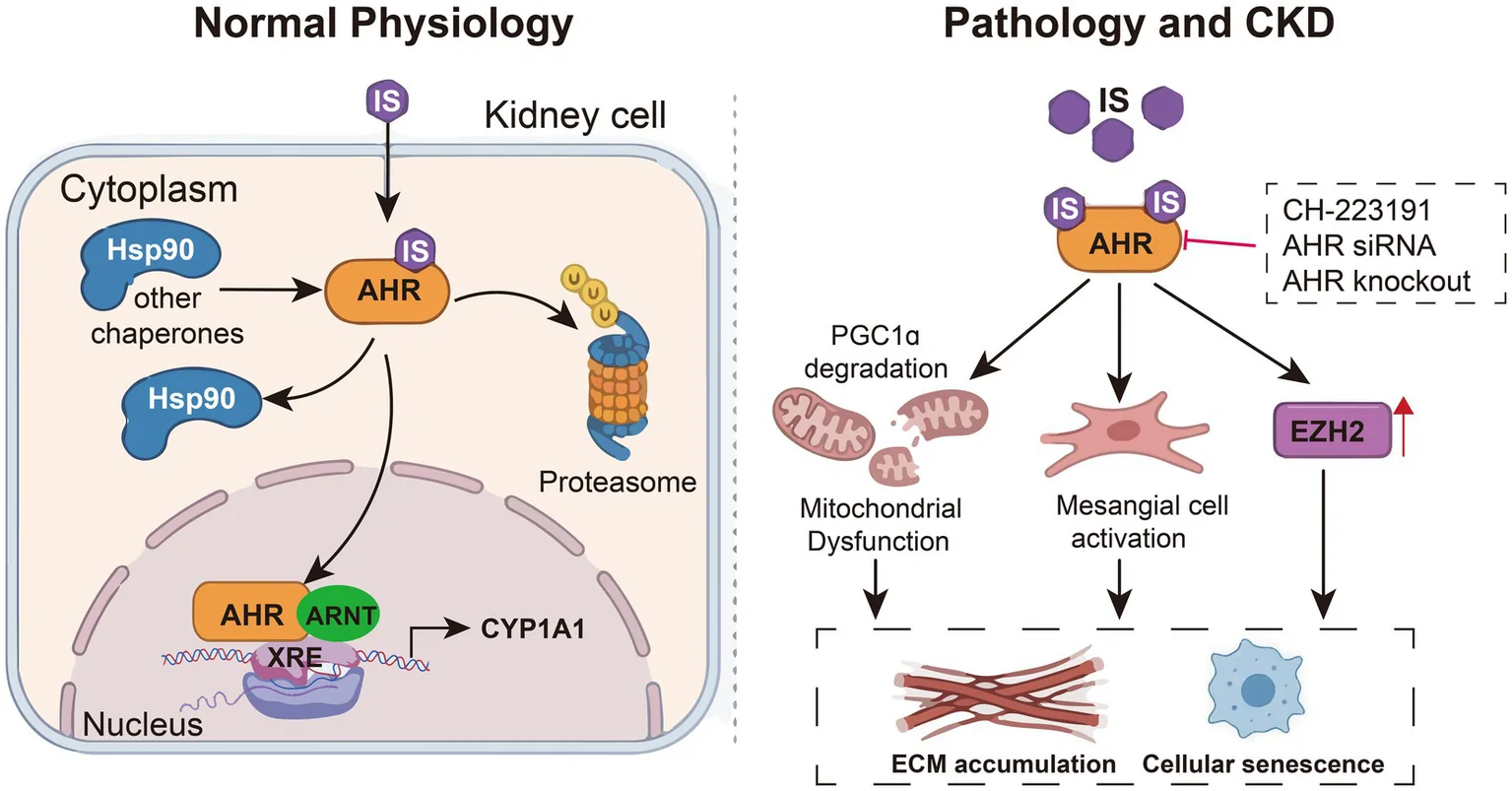
Mechanistic overview of IS-induced renal injury via the AHR signaling pathway. IS activates AHR, leading to downstream transcriptional responses and contributing to mitochondrial dysfunction, extracellular matrix accumulation, and cellular senescence in different renal cell types. Experimental inhibition or knockdown of AHR attenuates these IS-induced effects. AHR, aryl hydrocarbon receptor; ARNT, AHR nuclear translocator; CYP1A1, cytochrome P450 family 1 subfamily A member 1; ECM, extracellular matrix; EZH2, enhancer of zeste homolog 2; Hsp90, heat shock protein 90; IS, indoxyl sulfate; PGC-1α, peroxisome proliferator-activated receptor γ coactivator-1α; XRE, xenobiotic response element.

Overview, current evidence supports AHR signaling as an important upstream regulatory hub in IS-mediated renal injury. Its activation appears to link IS exposure with mitochondrial dysfunction, cellular senescence, inflammatory responses, and extracellular matrix remodeling in a cell-specific manner. However, the strongest evidence remains experimental, and the clinical relevance of AHR-targeted intervention requires further validation.

### 3.2 Oxidative stress and inflammation

Chronic hypoxia-induced tubulointerstitial microvascular injury is a common pathological mechanism underlying kidney disease progression (55, 56). Palm et al. (57) reported that IS significantly increases oxygen consumption in renal tubular cells by inducing oxidative stress, thereby exacerbating local hypoxia. Specifically, treatment of human proximal tubular cells with 0.05 mM IS increased oxygen consumption by approximately 140% compared with baseline levels (57). Notably, this effect was abolished following NADPH oxidase silencing (57). These findings identify NADPH oxidase-dependent ROS generation as an important early response to IS in renal tubular cells. On the one hand, IS directly promotes ROS generation through NADPH oxidase activation and subsequently activates the NF-κB and p53 signaling pathways (58). On the other hand, the IS–AHR complex upregulates NADPH oxidase isoform 4 (NOX4) and suppresses STAT3 signaling through enhanced phosphorylation of cAMP response element-binding protein (CREB) and NF-κB, thereby promoting excessive ROS production (59). Thus, AHR and NADPH oxidase/ROS signaling appear to be functionally interconnected, with AHR activation potentially amplifying the oxidative response to IS rather than acting as an isolated pathway.

IS not only directly damages renal tubular epithelial cells but also accelerates renal microvascular dysfunction through impairment of vascular endothelial function. Yu et al. (14) demonstrated that, in human vascular endothelial cells, IS enhances ROS production while reducing nitric oxide (NO) levels through activation of NADPH oxidase and/or the mitochondrial respiratory chain. This process inhibits endothelial cell proliferation and induces endothelial dysfunction, ultimately contributing to renal injury through microvascular rarefaction (14). The cell-specific findings therefore suggest that IS may promote tubular and microvascular injury through partially distinct but convergent oxidative mechanisms.

Furthermore, IS impairs the antioxidant defense capacity of renal tubular cells. Under physiological conditions, the Nrf2/Keap1 pathway serves as a central defense system against oxidative stress. Under normal conditions, Keap1 suppresses Nrf2 activity by promoting its cytoplasmic retention and degradation (60). Upon oxidative stress, Nrf2 is released from Keap1-mediated inhibition, translocates to the nucleus, and induces the expression of antioxidant genes, including HO-1, NQO1, and GCLC, thereby protecting cells against oxidative damage (60). The renoprotective role of the Nrf2/HO-1 axis in oxidative and inflammatory kidney injury is also supported by recent experimental evidence showing that enhancement of Nrf2/HO-1 signaling contributes to the protective effects of lixisenatide against diabetes-induced renal injury, with further amplification following ticagrelor co-administration (61).

However, experimental studies suggest that IS-activated AHR suppresses Nrf2 nuclear translocation and downstream antioxidant responses in renal tubular cells (62, 63). This may weaken the cellular antioxidant defense system, leading to depletion of endogenous antioxidants such as glutathione (GSH) and further accumulation of ROS (62, 63). Through these interconnected processes, excessive ROS accumulates within the kidney and subsequently activates pro-inflammatory signaling pathways, including NF-κB and MAPK, thereby inducing the release of inflammatory cytokines such as TNF-α, IL-1β, and IL-6 (64, 65). This establishes a mechanistic link between oxidative stress and inflammation, whereby IS-induced ROS activates pro-inflammatory signaling while impaired antioxidant defenses sustain ROS accumulation, creating a vicious cycle that amplifies renal inflammation and injury in CKD, as illustrated in Figure 2.

**Figure 2:**
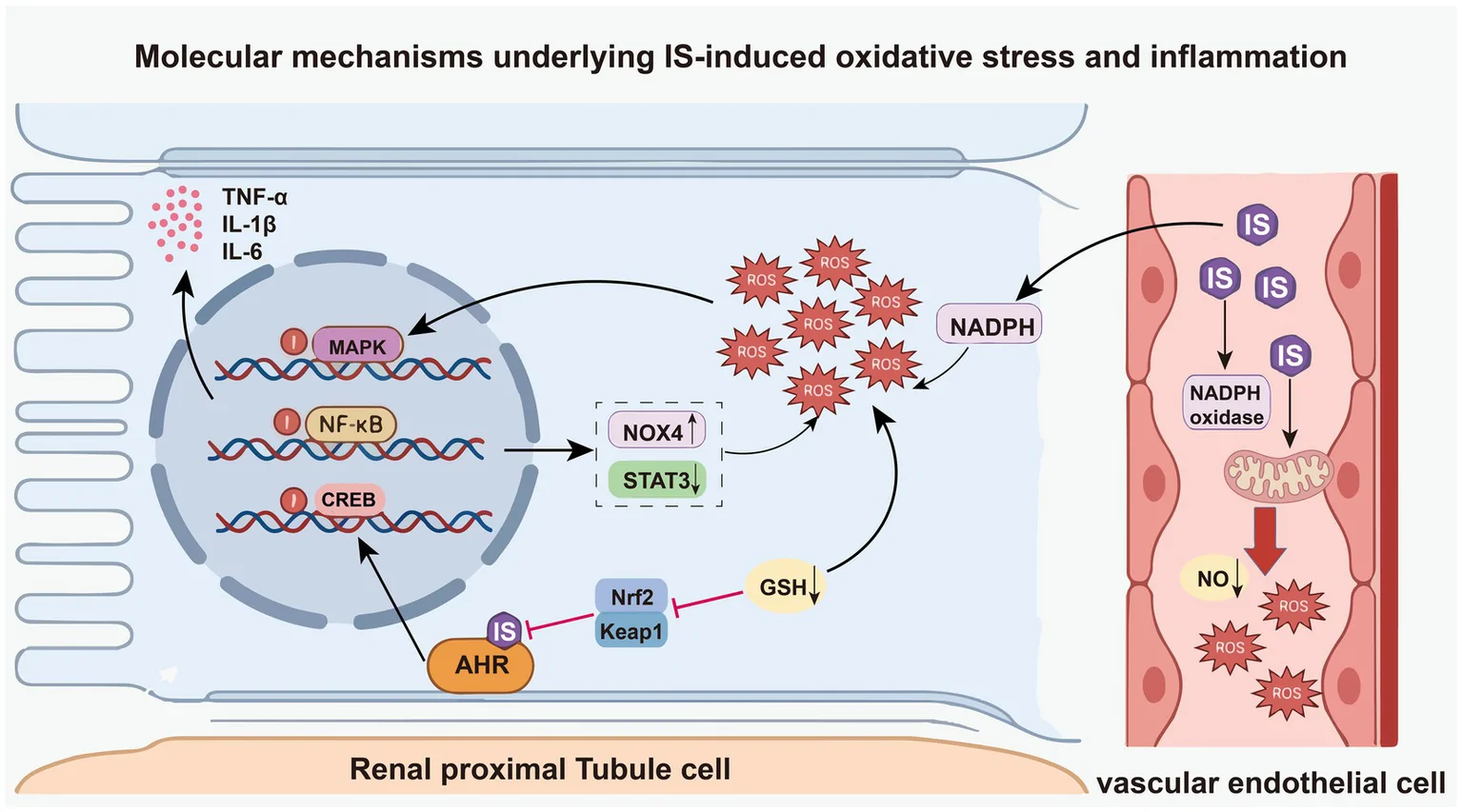
Oxidative stress and inflammation in IS-induced renal injury. IS promotes ROS production through NADPH oxidase activation and suppression of the Nrf2/Keap1 antioxidant defense system, thereby activating inflammatory pathways such as NF-κB and MAPK. The resulting oxidative stress and inflammation form a self-reinforcing cycle that contributes to renal injury. AHR, aryl hydrocarbon receptor; CREB, cyclic AMP response element-binding protein; GSH, glutathione; IL-1β, interleukin-1β; IL-6, interleukin-6; IS, indoxyl sulfate; Keap1, Kelch-like ECH-associated protein 1; MAPK, mitogen-activated protein kinase; NADPH, nicotinamide adenine dinucleotide phosphate; NF-κB, nuclear factor-κB; NO, nitric oxide; Nrf2, nuclear factor erythroid 2-related factor 2; NOX4, NADPH oxidase 4; ROS, reactive oxygen species; STAT3, signal transducer and activator of transcription 3; TNF-α, tumor necrosis factor-α.

Collectively, current evidence places oxidative stress and inflammation downstream of IS exposure and closely connected with AHR signaling. The NADPH oxidase/ROS axis, impaired Nrf2/Keap1 antioxidant defense, and subsequent activation of NF-κB and MAPK provide a mechanistic framework linking IS to tubular and microvascular injury. However, direct evidence for these pathway interactions is largely derived from experimental studies, and their relative contribution to human CKD progression remains to be determined.

### 3.3 Apoptosis, pyroptosis, and ferroptosis

Apoptosis, pyroptosis, and ferroptosis represent critical forms of cell death involved in IS-mediated renal injury. Rather than acting as completely independent pathways, these forms of regulated cell death appear to converge on several upstream disturbances, particularly ROS accumulation, mitochondrial dysfunction, inflammatory signaling, and impaired antioxidant capacity, as illustrated schematically in Figure 3.

**Figure 3:**
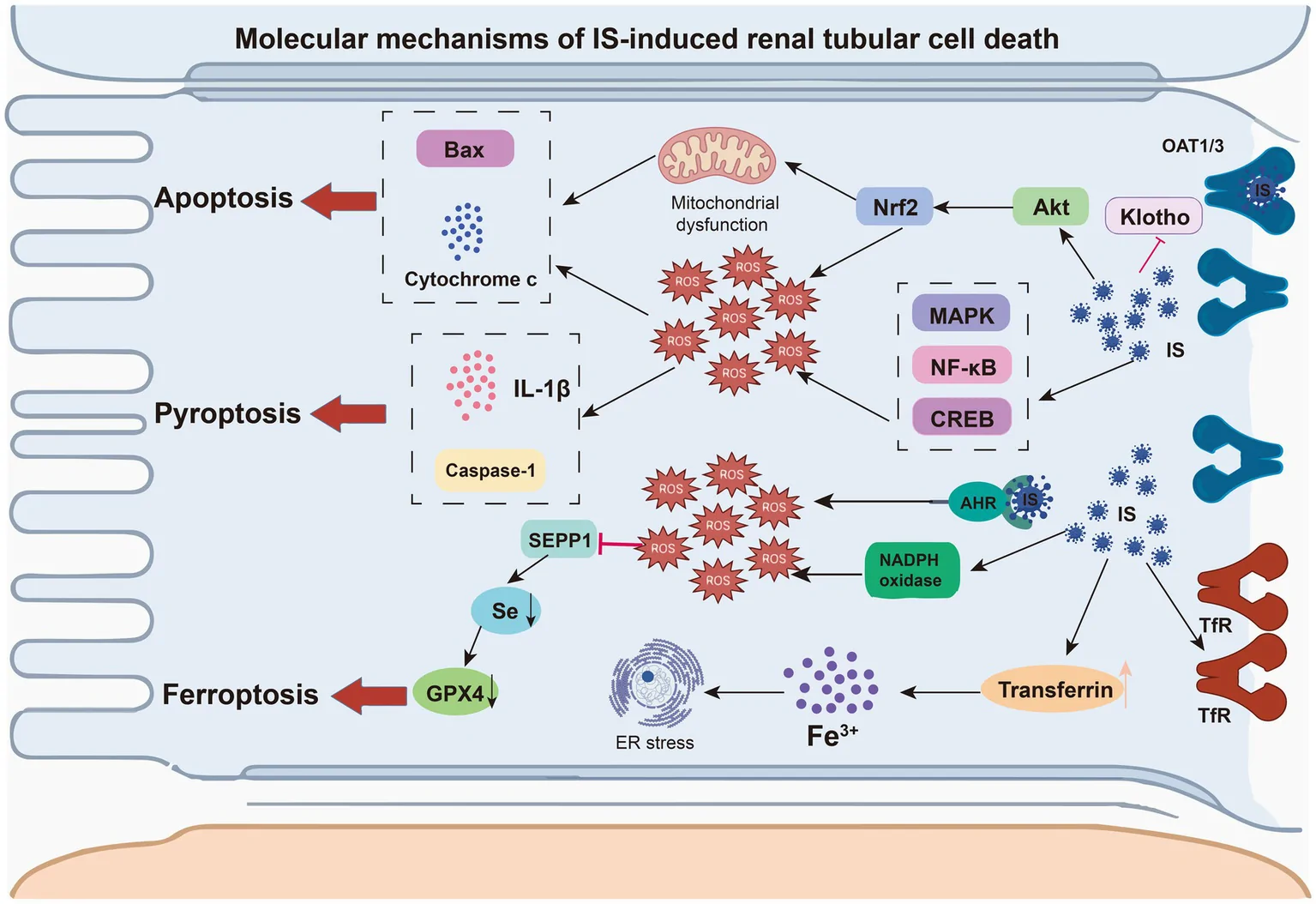
Multiple cell death pathways induced by indoxyl sulfate in renal cells. IS induces apoptosis, pyroptosis, and ferroptosis through mechanisms involving oxidative stress, inflammatory signaling, mitochondrial dysfunction, and dysregulated iron and selenium homeostasis. AHR, aryl hydrocarbon receptor; Akt, protein kinase B; Bax, Bcl-2-associated X protein; CREB, cyclic AMP response element-binding protein; GPX4, glutathione peroxidase 4; IL-1β, interleukin-1β; IS, indoxyl sulfate; MAPK, mitogen-activated protein kinase; NF-κB, nuclear factor-κB; Nrf2, nuclear factor erythroid 2-related factor 2; OAT, organic anion transporter; ROS, reactive oxygen species; SEPP1, selenoprotein P; TfR, transferrin receptor.

Accumulating evidence demonstrates that IS significantly enhances ROS production, induces the expression of the pro-apoptotic protein Bax, and promotes phosphorylation of MAPK, NF-κB p65, and Akt, ultimately resulting in apoptosis of HK-2 cells (14). In a subsequent study, Sun et al. (66) demonstrated that IS treatment induces apoptosis in HK-2 cells through inhibition of the Klotho/Akt/Nrf2 axis, leading to mitochondrial respiratory chain dysfunction and ROS accumulation. This process ultimately results in upregulation of Bax and cytochrome c, thereby inducing apoptosis (66). These *in vitro* findings support a role for oxidative and mitochondrial dysfunction in IS-induced tubular epithelial cell apoptosis. In addition, IS has been shown to bind to NIMA-related kinase 7 (NEK7) in vascular endothelial cells, thereby activating the NLRP3 inflammasome and caspase-1, which subsequently induce pyroptosis and contribute to vascular endothelial injury (67). This finding provides further insight into the molecular mechanisms underlying IS-mediated vascular endothelial injury in ESRD.

Regarding pyroptosis, Mihajlovic et al. (68) reported that IS stimulation of proximal tubular cells (ciPTEC-OAT1) activates the NF-κB signaling pathway, leading to increased ROS production and exacerbated oxidative stress. Consequently, caspase-1 activation and IL-1β production are enhanced, thereby aggravating inflammatory responses and inducing pyroptosis (68). This pathway provides a potential mechanistic connection between the oxidative and inflammatory responses described above and inflammatory cell death.

IS can also induce ferroptosis through dysregulation of iron metabolism, thereby accelerating renal fibrosis progression (69). In a related investigation, Cai et al. (70) reported that IS-exposed renal tubular cell cultures and adenine-induced CKD mouse models exhibited characteristic features of ferroptosis, including iron accumulation, impaired antioxidant defenses, elevated ROS levels, and increased lipid peroxidation products. IS has been shown to upregulate the expression of transferrin and transferrin receptors, thereby promoting intracellular iron accumulation, cellular senescence, and endoplasmic reticulum stress, ultimately contributing to renal injury and fibrosis (70). In another mechanistic study, Nakano et al. (71) demonstrated that IS enhances ROS production through the organic anion transporter/AHR/NADPH oxidase pathway and suppresses expression of the selenium transport protein selenoprotein P (SEPP1). Given that redox homeostasis in renal tubules is tightly regulated by the selenium-containing enzyme glutathione peroxidase 4 (GPX4), reduced selenium levels in CKD mouse kidneys lead to decreased GPX4 expression (71). These molecular alterations ultimately promote ferroptotic cell death, further linking the AHR/NADPH oxidase/ROS axis to impaired antioxidant defenses and ferroptosis.

These findings suggest that IS can induce apoptosis, pyroptosis, and ferroptosis through partially overlapping pathways involving ROS accumulation, inflammatory signaling, mitochondrial dysfunction, and impaired antioxidant defenses. However, direct clinical evidence remains limited, and the extent to which these forms of cell death independently or jointly drive CKD progression remains unclear.

### 3.4 Fibrosis

Renal fibrosis represents the final common pathological outcome of CKD, characterized by excessive extracellular matrix (ECM) deposition, aberrant myofibroblast activation, and transforming growth factor-β (TGF-β)-mediated epithelial–mesenchymal transition (EMT) in renal tubular epithelial cells (72). Clinical studies have demonstrated a positive association between serum IS levels and the severity of renal fibrosis in patients with CKD (73). Mechanistic studies in experimental models further support a potential causal role for IS in promoting renal fibrogenesis. Continuous intraperitoneal administration of IS (100 mg/kg/day for 4 weeks) in non-nephrectomized mice markedly induced renal fibrosis through activation of the Wnt/β-catenin signaling pathway (73). In 5/6 nephrectomized rat models, IS treatment upregulated the renal expression of profibrotic mediators, including TGF-β1, tissue inhibitor of metalloproteinase-1 (TIMP-1), and type I collagen, thereby accelerating renal fibrotic progression (74). *In vitro* studies further demonstrated that IS stimulation of HK-2 cells significantly increased the expression of fibrosis-related factors, including TGF-β, Smad2, Smad3, and Smad7, thereby promoting EMT (75). In mesangial cells, IS also promoted cell proliferation through activation of cyclooxygenase-2 (COX-2), an inducible inflammation-associated enzyme, thereby contributing to fibrogenesis (76).

These findings suggest that IS-associated fibrosis involves multiple renal cell types and signaling pathways rather than a single canonical pathway. Mechanistically, accumulating evidence indicates that IS mediates these profibrotic effects through multiple signaling pathways, as illustrated in Figure 4. IS induces STAT3 phosphorylation, thereby enhancing the expression of monocyte chemoattractant protein-1 (MCP-1), TGF-β1, and α-smooth muscle actin (α-SMA) (77). IS-induced ROS activate the NF-κB p65 subunit through phosphorylation, leading to increased p53 expression and accumulation, which subsequently promotes TGF-β1 secretion (78). IS also activates mTORC1 signaling in tubular epithelial cells via the OAT/NADPH oxidase/ROS pathway, resulting in reduced E-cadherin expression and increased α-SMA and COL1A1 levels, ultimately promoting renal fibrosis (79). Moreover, in murine mesangial cells, IS activates the ERK1/2 pathway through the (pro)renin receptor [(P)RR], thereby increasing the expression of fibrosis-associated mediators, including MMP9, TIMP1, collagen IV, TGF-β, and fibronectin, which further contribute to renal fibrogenesis (80).

**Figure 4:**
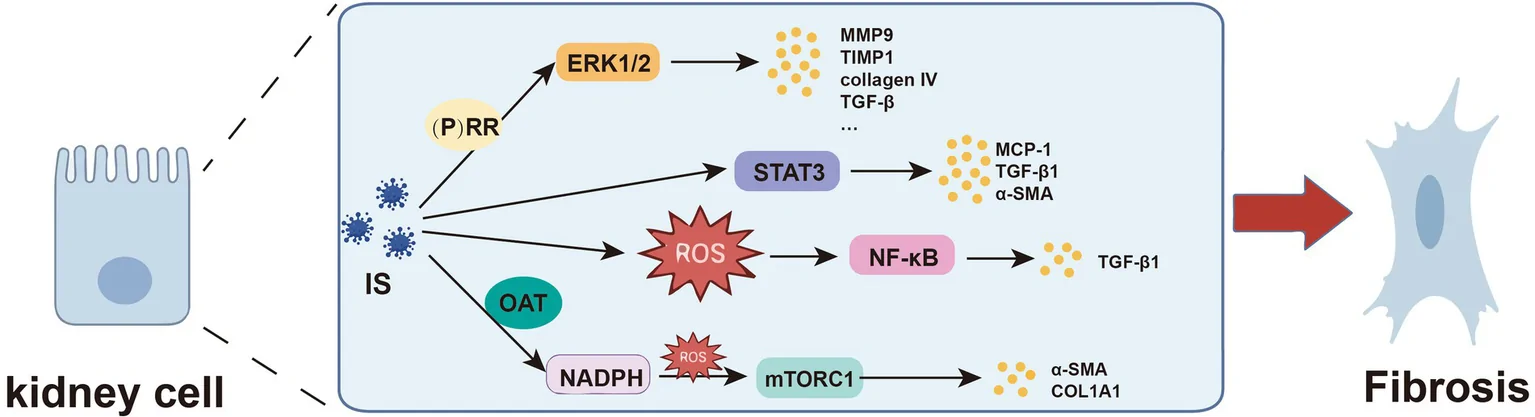
Signaling pathways involved in IS-induced renal fibrosis. IS activates multiple profibrotic pathways, including STAT3, NF-κB, TGF-β/Smad, mTORC1, and ERK1/2, leading to the development and progression of renal fibrosis.α-SMA, α-smooth muscle actin; COL1A1, collagen type I alpha 1 chain; IS, indoxyl sulfate; MCP-1, monocyte chemoattractant protein-1; MMP9, matrix metalloproteinase-9; mTORC1, mechanistic target of rapamycin complex 1; NF-κB, nuclear factor-κB; OAT, organic anion transporter; (P)RR, (pro)renin receptor; ROS, reactive oxygen species; STAT3, signal transducer and activator of transcription 3; TGF-β, transforming growth factor-β; TIMP1, tissue inhibitor of metalloproteinase-1.

At present, IS appears to promote renal fibrosis through multiple, potentially interconnected signaling pathways across different renal cell types, with these pathways converging on TGF-β1 signaling, EMT, and extracellular matrix accumulation. However, the causal relationships and crosstalk among these pathways, as well as their clinical relevance, remain to be clarified.

Overall, current evidence supports a hierarchical model of IS-mediated renal injury rather than a series of independent signaling events. Dietary and microbial metabolism generates IS, which accumulates in CKD and activates AHR as an important upstream regulatory hub. AHR signaling can interact with the NADPH oxidase/ROS axis and impair Nrf2/Keap1-mediated antioxidant defense, thereby promoting oxidative stress and activation of inflammatory pathways such as NF-κB and MAPK. These interconnected disturbances may subsequently trigger apoptosis, pyroptosis, and ferroptosis in a cell-specific manner and converge on profibrotic pathways involving STAT3, NF-κB, mTORC1, ERK1/2, and TGF-β/Smad signaling. The resulting tubular, endothelial, and mesangial cell injury may collectively contribute to extracellular matrix accumulation and CKD progression. However, the strength of evidence varies across these mechanisms: clinical and observational studies mainly support associations between IS exposure, AHR activity, and renal dysfunction, whereas causal and cell-specific mechanisms are supported predominantly by *in vitro* and animal studies. Accordingly, the proposed pathway hierarchy should be regarded as a mechanistic framework rather than a fully established sequence in human CKD, and further studies are needed to clarify pathway cross-talk, cell-specific contributions, and whether pharmacological disruption of these pathways can improve clinically meaningful renal outcomes.

## 4 Nutritional, microbiome-based, and IS-targeted therapeutic strategies

### 4.1 Dietary intervention and nutritional supplementation

As discussed above, dietary tryptophan serves as the primary source of IS. Therefore, regulating intake of dietary tryptophan constitutes a fundamental strategy for reducing serum IS concentrations (Table 1). Stanford et al. (81) evaluated dietary quality in hemodialysis patients and demonstrated that unhealthy dietary patterns, particularly excessive consumption of animal fats, sweets, and desserts, were associated with significantly increased serum total and free IS levels. In contrast, healthy dietary patterns have been shown to reduce IS concentrations in both healthy individuals and patients with CKD. Patel et al. (82) reported that long-term vegetarian diets significantly reduced mean IS excretion by 58% (95% CI, 39–71%) in healthy individuals. Rossi et al. (83) recruited 40 patients with CKD and found that the dietary protein-to-fiber ratio was positively correlated with total serum IS levels (*R* = 0.40, *p* = 0.012), independent of eGFR, sex, and diabetic status. McFarlane et al. (84) conducted a one-week dietary assessment in 68 patients with CKD and observed that individuals with higher dietary fiber intake exhibited significantly lower serum IS levels than those consuming other dietary patterns. Hansen et al. (85) implemented a one-week “New Nordic Renal Diet,” characterized by high fiber, low protein, and low phosphorus intake, in patients with CKD stages 3–4, resulting in a 29% reduction in 24 h urinary IS excretion (*p* < 0.001). Although these findings support a potential role for overall dietary patterns in modulating IS production, the short intervention periods and reliance on surrogate biochemical endpoints limit conclusions regarding sustained clinical benefit.

**Table 1:** Clinical evidence for therapeutic strategies targeting indoxyl sulfate.

Therapeutic strategy	Study design	Intervention/duration	Types of patients	Number of patients	Results	References
Dietary intervention	Cross-sectional study	N/A	CKD stages 4 to 5	40	The protein-fiber index was associated with total serum IS (*r* = 0.40, *p* = 0.012).	(83)
	Cross-sectional study	N/A	CKD (avg. eGFR 34 ± 11 mL/min/1.73 m^2^)	68	Dietary fiber intake was associated with lower levels of total indoxyl sulfate.	(84)
	Randomized controlled crossover trial	New Nordic Renal Diet, 1 week	CKD stages 3 to 4	18	24-h urine excretion of IS were reduced by 29% (*p* < 0.001).	(85)
	Cross-sectional Study	low protein diet (LPD, <0.8 g/kg body weight/day) vs. normal protein diet (NPD, 1 g/kg body weight/day), 3 months	CKD (avg. eGFR 31.95 ± 24.31 mL/min/1.73 m^2^)	43 (LPD, *n* = 16; NPD, *n* = 27)	The serum concentration of IS (total form, *p* = 0.054; free form, *p* = 0.105) did not differ significantly between LPD vs. NPD.	(89)
Synbiotics	Randomized controlled trial	*Lactobacillus acidophilus*, *Lactobacillus casei*, and *Bifidobacterium lactis*, 32 billion colony forming units per day plus 3.2 g of inulin, 12 weeks.	CKD (avg. eGFR 15 to 45 mL/min/1.73 m^2^)	17	Reduced serum level of IS (21.5% vs. 5.3%, *p* < 0.001).	(95)
	Prospective, non-randomized, placebo-controlled study	Capsules containing *Lactobacillus acidophilus* La-14 (2 × 10^11^ CFU/g) + fructooligosaccharides, 10 months	CKD stages 4 to 5	21	Treatment showed greater IS reduction (−0.0154 ± 0.0082 ng/mL vs. −0.0031 ± 0.0065 ng/mL, *p* < 0.001).	(96)
	Uncontrolled prospective single-arm study	*Lactobacillus acidophilus* La-14 2 × 10^11^ (CFU)/g and prebiotic fructooligosaccharides, 8 weeks.	ESRD	30	A significant decrease in the parameters compared to the baseline IS (212.89 ± 208.59 ng/mL vs. 47.08 ± 3.24 ng/mL, *p* < 0.001).	(97)
AST-120	Randomized controlled trial	3 doses of AST-120 (0.9, 2.1, or 3.0 g) 3 times daily for 12 weeks.	CKD(avg. SCr 265 to 530 μmol/L)	164	Serum IS levels decreased in a dose-dependent manner: 39.3% (9.0 g), 21.1% (6.3 g), and 2.6% (2.7 g) at week 12, compared to baseline.	(112)
Hemodialysis	Randomized controlled trial	A DVB-PVP cartridge and a symbiotic NATUREN G®, 8 weeks.	ESRD	16	Total IS plasma levels decreased 31% (*p* = 0.04) from baseline.	(123)
	Randomized controlled trial	HAHD using the novel pHA130 cartridge, 8 weeks.	ESRD	30	The combined HAHD group demonstrated a significantly greater IS reduction than the HDF-q2w group (46.9% vs. 31.8%; *p* = 0.044).	(129)
Traditional Chinese Medicine	Prospective, non-randomized controlled trial	High-position colon dialysis combined with TCM retention enemas (administered at least 12 times every other day).	Non-dialysis patients with CKD stages 3–5	159	IS levels decreased in both groups; significant reduction in treatment group (*p* < 0.05).	(137)

AST-120, an oral carbon adsorbent; CFU, colony-forming units; CKD, chronic kidney disease; DVB-PVP, divinylbenzene–polyvinylpyrrolidone; eGFR, estimated glomerular filtration rate; ESRD, end-stage renal disease; HAHD, hemoadsorption combined with hemodialysis; HDF, hemodiafiltration; IS, indoxyl sulfate; SCr, serum creatinine; TCM, traditional Chinese medicine; N/A, not applicable.

Increased dietary fiber supplementation has likewise been shown to effectively reduce IS levels. A meta-analysis conducted by Wathanavasin et al. (86) revealed that dietary fiber supplementation significantly decreased serum IS concentrations (SMD − 0.34, 95% CI − 0.57 to −0.12; *p* < 0.01), and this effect appeared to be minimally affected by study design, ethnicity, CKD status, risk of bias, fiber type, dosage, solubility, or supplementation duration. The underlying mechanism may involve reduced protein delivery to the colon and increased availability of fermentable substrates for gut microbiota under high-fiber dietary conditions, thereby promoting amino acid utilization (82). Nevertheless, the clinical significance of this reduction remains uncertain because most available studies have focused on circulating or urinary IS rather than hard renal outcomes. Thus, increasing dietary fiber intake may currently represent one of the more consistently supported nutritional approaches for reducing IS exposure, provided that dietary modification is compatible with the individual patient’s nutritional and metabolic requirements.

Given that dietary protein is the principal source of tryptophan, restricting protein intake may directly reduce the amount of tryptophan delivered to the colon, thereby decreasing IS production. However, whether low-protein diets can effectively reduce IS concentrations remains controversial. Several studies have supported the beneficial effects of dietary protein restriction. For instance, a randomized controlled trial conducted by Poesen et al. (87) in healthy individuals demonstrated that serum IS levels and 24 h urinary IS excretion were significantly higher in the high-protein diet group than in the low-protein diet group (*p* = 0.004 and *p* = 0.001, respectively). A clinical study conducted by Marzocco et al. (88) further demonstrated that, compared with a low-protein diet (40–60 g/day), a very low-protein diet (30–35 g/day) reduced serum IS concentrations by approximately 35%. Nevertheless, conflicting findings have also been reported. Wu et al. (89) divided participants into low-protein diet CKD, normal-protein diet CKD, and non-CKD control groups and observed no significant differences in serum total or free IS levels between CKD patients receiving low-protein and normal-protein diets. Similarly, Tsai et al. (90) conducted a one-week therapeutic dietary intervention in hemodialysis patients characterized by adequate caloric and protein intake together with high dietary fiber content and observed only a trend toward reduced IS levels (*p* = 0.07). These inconsistencies suggest that protein quantity alone may not adequately predict IS generation. Instead, protein source, dietary fiber availability, the protein-to-fiber ratio, and interindividual differences in gut microbial metabolism may jointly determine systemic IS exposure. To further address this issue, Lin et al. (17) refined an oral tryptophan challenge test in healthy individuals and demonstrated marked interindividual variability in IS production, which may be attributable to differences in gut microbiota composition and bacterial tryptophanase activity. These findings provide a rationale for a precision-nutrition approach in which dietary recommendations are tailored according to microbial metabolic characteristics and individual nutritional status rather than uniformly imposing protein restriction.

From a clinical perspective, long-term dietary adherence and nutritional safety remain important considerations. Excessive protein restriction may contribute to malnutrition and muscle mass loss, thereby adversely affecting the overall health status of patients with CKD (91). Moreover, dietary recommendations must be balanced against potassium, phosphorus, energy, and overall protein requirements according to CKD stage and dialysis status. Therefore, future studies should determine whether specific dietary patterns, rather than isolated nutrient restriction, can sustainably modify gut microbial tryptophan metabolism and improve clinically meaningful outcomes. Collectively, current evidence supports dietary pattern modification and adequate dietary fiber intake as potentially useful approaches for reducing IS exposure, whereas the benefits of protein restriction remain less consistent. Importantly, the available evidence primarily supports biochemical effects on IS rather than definitive improvements in CKD progression or patient-centered outcomes. A precision-nutrition strategy integrating dietary composition, gut microbial characteristics, and individual nutritional status may therefore provide a more rational framework for future IS-targeted nutritional interventions.

### 4.2 Gut microbiota-based interventions

Gut microbial dysbiosis is increasingly recognized as an important determinant of intestinal tryptophan metabolism and circulating IS exposure in CKD. Fecal samples from patients with ESRD exhibit an increased abundance of bacterial families containing tryptophanase-producing species, including *Clostridiaceae*, *Enterobacteriaceae*, and *Verrucomicrobiaceae* (92). These bacteria metabolize tryptophan into indole, which is subsequently converted into IS in the liver and constitutes a major source of circulating IS. Accordingly, modulation of the gut microbiota has emerged as a promising therapeutic strategy for alleviating IS-related toxicity. This metabolic link provides a mechanistic basis for targeting the gut microbiota to limit indole generation and systemic IS exposure. Although the contribution of individual microbial taxa to IS production remains incompletely defined and may be influenced by CKD stage, diet, medications, and dialysis status, these observations support the therapeutic potential of microbiota modulation. Accordingly, current research has increasingly focused on restoring gut microbial homeostasis through microbiota-targeted interventions, including probiotics, prebiotics, synbiotics, and fecal microbiota transplantation (FMT).

Probiotics are beneficial microorganisms, including *Lactobacilli* and *Bifidobacteria*, that can modulate gut microbial composition upon administration, thereby reducing the production of gut-derived uremic toxins (93). Prebiotics are non-digestible carbohydrates that promote host health by selectively stimulating the growth and/or activity of specific beneficial bacteria within the colon (93). The combined administration of probiotics and prebiotics, termed synbiotics, has demonstrated particularly promising synergistic effects in clinical studies (Table 1). Jerez-Morales et al. (94) administered synbiotics to CKD rats and observed that, compared with baseline, serum IS levels decreased by 0.8% in the treatment group, whereas they increased by 38.8% in the control group. In a clinical trial, Mitrović et al. (95) administered synbiotics to CKD patients for 12 weeks and demonstrated that, compared with the placebo group, the intervention significantly improved gut microbiota composition, reduced serum IS concentrations by 21.5% (−21.5% vs. 5.3%, *p* < 0.001), and substantially attenuated the decline in eGFR. Kuskunov et al. (96) administered synbiotics to CKD patients for 10 months and reported significantly greater reductions in eGFR decline and serum IS levels compared with the placebo group (−0.0154 ± 0.0082 ng/mL vs. −0.0031 ± 0.0065 ng/mL, *p* < 0.001). In dialysis patients, 8 weeks of synbiotic administration significantly decreased IS levels compared with baseline values (212.89 ± 208.59 ng/mL vs. 47.08 ± 3.24 ng/mL, *p* < 0.001) (97). In addition, several meta-analyses and network meta-analyses have reported reductions in IS concentrations following probiotic, prebiotic, or synbiotic interventions (98–101). Nevertheless, these findings should be interpreted cautiously. The available clinical studies are heterogeneous with respect to probiotic strains, prebiotic substrates, intervention duration, patient characteristics, and background dietary management. More importantly, most trials primarily assessed biochemical endpoints such as serum or urinary IS, while evidence for effects on renal outcomes, cardiovascular events, hospitalization, or survival remains limited. Accordingly, microbiota-targeted supplementation currently supports modulation of IS-related biomarkers, but not definitive clinical benefit.

FMT is a therapeutic strategy in which fecal material from a healthy donor is administered to a recipient through the gastrointestinal tract to correct gut microbial dysbiosis (102). By directly transferring a complex microbial community, FMT represents a more direct approach to restructuring the gut microbial ecosystem than probiotics or synbiotics. Recent studies have demonstrated that FMT not only improves gut microbial composition in patients with kidney disease (103, 104) but may additionally mitigate the nephrotoxic effects of PBUTs through restoration of intestinal barrier integrity and metabolic homeostasis (105). These effects may contribute to reduced generation or systemic exposure of PBUTs, including IS. Current research suggests that FMT may reduce serum IS levels in CKD (Table 2). Lauriero et al. (106) administered FMT in a humanized mouse model of IgA nephropathy and observed a significant reduction in fecal indole, accompanied by attenuation of proteinuria. Liu et al. (107) transplanted fecal microbiota from sham-operated rats into 5/6 nephrectomized CKD rats, resulting in normalization of gut microbiota composition, decreased serum levels of IS, p-cresol sulfate (PCS), improvements in serum creatinine (SCr) and blood urea nitrogen (BUN), and amelioration of renal histopathological changes. Zhong et al. (108) transplanted washed microbiota from healthy donors into patients with CKD and reported that the intervention group exhibited significantly improved SCr, BUN, and eGFR compared with the non-transplanted control group, resulting in a shift of the recipients’ gut microbiota toward that of healthy donors and enhanced urinary indole excretion. These findings suggest that FMT may modulate gut microbial composition and tryptophan metabolism, potentially reducing intestinal indole production and systemic exposure to IS and other PBUTs. Nevertheless, the evidence remains preliminary, with substantial heterogeneity in donor and recipient characteristics, transplantation protocols, and outcome measures, while clinical studies are limited by small sample sizes and insufficient long-term follow-up. Potential safety concerns, including transmission of infectious agents and unintended transfer of microbial traits, further highlight the need for rigorous donor screening, standardized preparation procedures, and long-term safety monitoring (109). Well-designed randomized controlled trials are needed to determine whether FMT can achieve sustained reductions in IS exposure and translate these effects into clinically meaningful renal benefits.

**Table 2:** Preclinical evidence for therapeutic strategies targeting indoxyl sulfate.

Therapeutic strategy	Study design	Intervention/duration	Types of model	Results	References
Synbiotics	Animal experiment	The Synbiotic *Lactobacillus bulgaricus* 6c3 Strain, Inulin and Fructooligosaccharide, 10^6^ CFU/g, 16 weeks.	5/6 nephrectomy rats	The synbiotic group reduced IS by 0.8% while the nephrectomized untreated animals increased it by 38.8%.	(94)
	Animal experiment	Fecal microbiota transplantation with no nephrectomy (Sham), 3 weeks.	5/6 nephrectomy rats	FMT significantly reduced the accumulation of PBUTs, including IS.	(107)
Hemodialysis	*In vitro*	A simulated hemodialysis session was performed using bovine blood spiked with PBUTs in the presence of various medium chain fatty acids.	N/A	The per-dialytic infusion of octanoate significantly improved the fractional removal of IS from 36 to 91%.	(120)
	*In vitro*	Established a macro-dialysis and added PCD1 obtained from 2 h crosslinking to the dialysate.	N/A	PCD1 doubled IS removal from the dialysate compared with removal without PCD1.	(122)
	*In vitro*	Introduction of activated carbon into the dialysate stream.	N/A	Increased the clearance of the protein-bound solutes IS by 77% ± 12%.	(121)
	*In vitro*	DVB-PVP resin cartridge; 1, 3, and 6 h perfusion.	N/A	After 6 h of perfusion, DVB-PVP removed approximately 54% of total IS.	(123)
	*In vitro*	NU-1000 particles (NU@F)	N/A	The removal efficiency for IS was 65.0%.	(124)
	*In vitro*	A class of cationic polymeric networks, denoted as CPN-X6-CPN-X9.	N/A	CPN-X9 achieved up to 93% IS removal under recycling in vitro dialysis.	(125)
	*In vitro*	A tailored hemoperfusion adsorbent by compounding SPSf and PEI into PES microspheres.	N/A	In a simulated hemoperfusion setting, the removal ratio of IS by H-PES/SPSf@PEI microspheres reaches nearly 75.33%.	(127)
	*In vitro*	Ibuprofen-immobilized CA-based thin films.	N/A	Competitive displacement binding assays 1.2-fold decrease in the total percentage of IS, and the free percentage of IS increased 1.3 to 3.0 times.	(128)
Traditional Chinese Medicine	Animal experiment	Fushen Granule, 6 weeks.	5/6 nephrectomy rats	Compared with the model group in the same period, Fushen Granule at high doses rats had a decreased abundance of IS (6.35 ± 2.24 vs. 3.19 ± 1.56, *p* < 0.05).	(130)
	Animal experiment	Bupi Yishen formula (15 or 30 g/kg/day), 6 weeks.	Mice with adenine-induced CKD.	High-dose BYF showed lower IS levels compared to model (*p* < 0.01).	(134)
	Animal experiment	Tangshen Formula, 12 weeks.	Streptozotocin injection and uninephrectomy-induced diabetic nephropathy rat model.	Tangshen Formula showed lower IS levels compared to model (*p* < 0.05).	(135)

BYF, Bupi Yishen Formula; CA, cellulose acetate; CFU, colony-forming units; CKD, chronic kidney disease; CPN, cationic polymeric network; DVB-PVP, divinylbenzene–polyvinylpyrrolidone; FMT, fecal microbiota transplantation; IS, indoxyl sulfate; PCD1, poly-β-cyclodextrins; PBUTs, protein-bound uremic toxins; PEI, polyethyleneimine; PES, polyethersulfone; SPSf, sulfonated polysulfone; N/A, not applicable.

Collectively, microbiota-targeted interventions provide a biologically plausible means of reducing IS exposure by modifying intestinal tryptophan metabolism. Current evidence is most supportive of reductions in circulating IS and related metabolic markers, whereas evidence for long-term renal protection remains insufficient. Future studies should move beyond taxonomic descriptions toward functional characterization of microbial tryptophan metabolism and should integrate dietary factors, microbial function, and host characteristics to identify patients most likely to benefit from microbiome-targeted interventions.

### 4.3 Intestinal adsorbent therapy: AST-120

AST-120 is particularly relevant to the gut–kidney axis because it acts within the gastrointestinal tract to adsorb indole, thereby interrupting the intestinal precursor supply of IS. AST-120 is an oral spherical carbon adsorbent composed of porous carbon particles with diameters of 0.2–0.4 mm that are insoluble in water and has been administered long-term to patients with CKD for over 20 years (110). One of its primary mechanisms of action is the adsorption of uremic toxins and their precursors in the gastrointestinal tract, thereby preventing their reabsorption into the bloodstream and promoting their elimination through feces (110). Niwa et al. (111) conducted a clinical trial in 1997, demonstrating that IS accumulation was associated with CKD progression and that AST-120 administration significantly reduced serum and urinary IS levels. Mechanistically, AST-120 adsorbs indole, the precursor of IS, in the gastrointestinal tract, thereby reducing its systemic absorption and subsequent hepatic conversion to IS (112) (Table 1). Consistent with this mechanism, a multicenter randomized controlled trial in patients with moderate to severe CKD showed that 12 weeks of AST-120 treatment reduced serum IS levels by 39.3 and 2.6% at doses of 9.0 and 2.7 g/day, respectively (112) Meta-analyses indicate that administration of AST-120 in CKD patients at stages 3–5 resulted in a reduction of serum IS levels by 2,800 ng/mL compared with baseline (95% CI: −0.46 to −0.11) (113). However, whether reductions in IS levels translate into meaningful clinical benefits remains uncertain. A retrospective analysis conducted in South Korea showed that AST-120 treatment not only reduced IS concentrations but also delayed CKD progression and the occurrence of adverse cardiovascular events (114). Conversely, the Evaluating Prevention of Progression in CKD trials, two large-scale, multinational, randomized, double-blind placebo-controlled studies, failed to demonstrate that AST-120 significantly delayed CKD progression or improved renal outcomes (115), despite its ability to reduce IS accumulation. These discrepant findings highlight the uncertainty regarding whether AST-120-induced reductions in IS translate into clinically meaningful renal or cardiovascular benefits.

Overall, current evidence supports the capacity of AST-120 to reduce systemic IS exposure through intestinal adsorption of indole. However, whether this biochemical improvement can be translated into long-term renal protection remains uncertain. In addition, preclinical and clinical studies suggest that AST-120 may alleviate several IS-associated pathological processes, including oxidative stress, inflammatory responses, endothelial dysfunction, renal injury, and musculoskeletal complications (116). Further prospective studies are required to clarify its clinical benefits and define its optimal role in CKD management.

### 4.4 Protein-binding-targeted dialysis and adsorption strategies

Although hemodialysis is the primary route for removing uremic toxins in patients with ESRD, PBUTs such as IS are tightly bound to HSA and challenging to remove effectively with conventional dialysis techniques. To address this clinical challenge, researchers have explored innovations in two main directions: replacement agents and adsorption membranes, with the goal of markedly improving IS clearance (117, 118).

In the replacement agent strategy, studies have focused on adding competitive binding agents or adsorbents to disrupt the IS-albumin interaction or directly capture IS (Tables 1, 2). Tao et al. (119) innovatively added albumin-binding competitors, including tryptophan or docosahexaenoic acid, to the blood circuit upstream of a dialysis device. Competitive displacement significantly increased IS removal rates from 10.2 to 18.5 and 27.7%, respectively (*p* < 0.001) (119). Similarly, in simulated hemodialysis, the addition of octanoate, a medium-chain fatty acid (MCFA) and HSA ligand, increased IS clearance from 36 to 91% (120). Li et al. further reported that poly-β-cyclodextrins added to the dialysate approximately doubled IS clearance in simulated dialysis, while activated carbon achieved an IS removal rate of 77% ± 12% in an *in vitro* dialysis model (121, 122). Rocchetti et al. (123) employed divinylbenzene-polyvinylpyrrolidone, a novel adsorbent resin, as a dialysis filter. *In vitro* experiments demonstrated that 54% of IS was removed on average after 6 h of perfusion, whereas *in vivo* experiments showed a 31% reduction post-dialysis (123). Guerrero et al. (124) incorporated perfluorotitanium into a zirconium-based metal–organic framework to produce fluorinated NU-1000 particles as dialysis adsorption filters. Simulated dialysis using patients’ ultrafiltrate samples showed an effective IS removal rate of 65.0% (124). Zhang et al. (125) reported cationic polymer network materials (CPN-X6–CPN-X9), with CPN-X6 demonstrating a maximum IS adsorption capacity of 1028.4 mg/g, and *in vitro* circulation dialysis achieved an IS removal rate up to 93%, indicating promising clinical applicability. Although these findings demonstrate the feasibility of enhancing IS removal, the reported clearance rates were obtained predominantly under experimental or simulated conditions and therefore cannot be directly extrapolated to clinical efficacy.

In the field of adsorption membrane technologies, researchers have focused on developing composite membranes possessing tailored affinity for efficient IS capture (Table 2). Lu et al. (126) developed polyethersulfone–zeolite composite membranes, which can adsorb approximately 550 μg of IS per gram of material, demonstrating high adsorption capacity. Wang et al. (127) covalently bonded sulfonated polysulfone and polyethyleneimine onto polyethersulfone microspheres to create a novel hemoperfusion adsorbent, which efficiently captured PBUTs through electrostatic interactions. Simulated blood perfusion experiments achieved an IS removal efficiency of 75.33% (127). Rodrigues et al. (128) leveraged the high affinity of ibuprofen for HSA to develop cellulose acetate membranes containing 25 wt% immobilized ibuprofen. Simulated dialysis showed that, compared with conventional membranes, total IS levels decreased by 1.2-fold, free IS levels increased 1.3–3.0-fold, and potential side effects from direct ibuprofen use were avoided (128). Collectively, these experimental membrane systems provide proof-of-concept evidence for improving PBUT clearance but remain largely preclinical.

Compared with these experimental approaches, pHA130 currently provides more direct clinical evidence. In a clinical trial, Yu et al. (129) used a novel adsorbent, pHA130, which competitively dissociates PBUTs from albumin and subsequently captures them. After 8 weeks, the intervention significantly reduced IS levels in patients. However, evidence remains limited, and whether this biochemical reduction translates into sustained improvements in renal function, cardiovascular outcomes, or quality of life requires further investigation. Overall, replacement agents and advanced adsorption membranes represent promising approaches for enhancing IS removal during extracorporeal blood purification, but most available evidence remains at the *in vitro* or preclinical stage. Further large-scale, multicenter clinical studies are needed to establish their long-term safety, clinical efficacy, and cost-effectiveness and to determine whether enhanced IS removal ultimately improves patient outcomes.

### 4.5 Traditional Chinese medicine (TCM)

TCM-based interventions represent an emerging multi-target approach within the gut–kidney axis, particularly because many formulations may simultaneously influence gut microbial composition, intestinal barrier function, inflammation, oxidative stress, and IS-related signaling pathways (Tables 1, 2). In animal models, Fushen Granule exhibited significant renal protective effects in 5/6 nephrectomy-induced CKD rats, markedly reducing SCr, BUN, uric acid (UA), and levels of IS and PCS (130). Mizhuo Guanchangye was administered via enema to adenine-induced CKD rats, significantly decreasing serum IS, lipopolysaccharide (LPS), IL-6, and TNF-α levels, while improving renal fibrosis and intestinal barrier function (131). The Huang Gan formula can mitigate systemic inflammation by inhibiting NF-κB signaling and concomitantly reduce uremic toxins such as IS and PCS (132). Yishen-Qingli-Huoxue formula significantly reduces serum IS levels and suppresses downstream AHR/Snai1 pathway activation, consequently ameliorating renal fibrosis (133). Bupi Yishen formula treatment in adenine-induced CKD mice enriched beneficial bacteria such as *Akkermansia muciniphila* and *Barnesiella intestinihominis*, improved intestinal barrier integrity, and significantly lowered serum IS, LPS, IL-1β, and TNF-α levels, thereby slowing CKD progression (134). Zhao et al. (135) treated DKD rats with Tangshen Formula, which alleviated renal inflammation by restoring gut microbiota balance, reducing levels of IS, endotoxin, and LPS, and inhibiting AHR, TLR4, NF-κB, and TNF-α expression. These preclinical findings provide mechanistic support for the potential of TCM to regulate the gut–microbiome–IS–kidney axis, but their translation to human CKD remains uncertain because animal models, formulations, dosages, and treatment durations differ substantially from clinical practice.

Beyond orally administered herbal formulations, recent evidence suggests that external TCM therapies, including acupuncture, tuina, bloodletting, and topical herbal applications, may also influence metabolic and renal-related processes by promoting metabolite excretion and modulating inflammatory and oxidative stress responses (136). Although this evidence is primarily derived from hyperuricemia and other metabolic disorders rather than IS-related CKD, it provides broader mechanistic support for the potential of TCM-based approaches to regulate renal excretory function and systemic inflammatory and oxidative stress pathways. A limited number of clinical studies have also reported reductions in serum IS following TCM-based interventions. Following oral administration of compound formulations combined with high colonic dialysis enemas, the treatment group showed significant reductions in serum IS levels, along with a decrease in *Escherichia coli* and an increase in *Bifidobacterium* and *Lactobacillus* abundance (137). In peritoneal dialysis patients, administration of Shen-Suining Granules led to a significant reduction in total serum IS levels compared with the control group (138). However, these clinical observations are based on a limited number of studies, and the available evidence is insufficient to determine whether reductions in IS translate into sustained improvements in renal or patient-centered outcomes.

In addition to compound formulations, monomers of Chinese traditional herbs have demonstrated potential for mitigating IS-related renal injury. Extracts of Lindera aggregata have been shown to inhibit IS-induced expression of Smad2, Smad3, Smad7, and TGF-β in HK-2 cells, thereby demonstrating antifibrotic effects (75). Astragaloside IV significantly suppressed IS-mediated oxidative stress and AHR activation, effectively preventing damage to renal tubular epithelial cells (139, 140). Ethanol extracts of *Salvia miltiorrhiza* markedly improved SCr, BUN, urinary protein, and IS levels, and suppressed IS-mediated EMT in adenine-induced CKD rat models (141). These findings further support the biological plausibility of targeting multiple IS-related pathways, but evidence from cell and animal studies cannot be considered equivalent to clinical efficacy.

The available evidence suggests that TCM may offer a multi-target strategy for modulating the gut microbiota and downstream inflammatory, oxidative, and fibrotic pathways associated with IS. Nevertheless, compared with more established dietary and microbiota-targeted approaches, evidence supporting the clinical efficacy of TCM for sustained IS reduction and renal protection remains limited and heterogeneous, with much of the mechanistic evidence derived from cell and animal studies. Interpretation is further complicated by substantial variability in herbal composition, preparation methods, active constituents, dosages, and treatment duration, while potential herb–drug interactions and insufficient long-term safety data remain important considerations. Future clinical studies should therefore prioritize standardized formulations and quality-control procedures, appropriate comparator groups, adequate sample sizes, clinically meaningful renal and patient-centered outcomes, and systematic safety monitoring to determine the efficacy, safety, and reproducibility of TCM-based IS-targeted interventions in patients with CKD.

### 4.6 Limitations, safety, and translational challenges of IS-targeted strategies

Despite the growing number of IS-targeted strategies, the available evidence remains heterogeneous in study design, intervention characteristics, and clinical endpoints. Much of the current evidence is derived from mechanistic studies, animal models, or small clinical trials, while long-term randomized evidence linking IS reduction to renal, cardiovascular, or patient-centered outcomes remains limited. Thus, reductions in circulating IS should not be interpreted as direct evidence of clinical benefit.

Safety and feasibility also require consideration when translating these approaches into clinical practice. Dietary interventions must balance IS reduction with adequate nutritional intake, whereas microbiota-based therapies, particularly FMT, require careful assessment of infection risk, donor and protocol variability, and long-term microbiome stability. For dialysis-based approaches, the safety of competitive binding agents and adsorption materials, including potential effects on other albumin-bound substances, remains insufficiently characterized. TCM-based interventions additionally face challenges related to formulation standardization, herb–drug interactions, and long-term safety.

Overall, the major challenge is to determine whether effective IS lowering can be translated into sustained clinical benefit without compromising nutritional status or treatment safety. Future research should therefore emphasize standardized interventions, adequately powered randomized controlled trials, longer follow-up, and clinically meaningful renal and cardiovascular outcomes to establish the therapeutic value of IS-targeted strategies in CKD.

## 5 Limitations and future perspectives

Although IS-targeted interventions have shown promising potential, several challenges continue to limit their clinical application. (i) Evidence quality and long-term clinical outcomes. Most clinical trials have involved relatively small sample sizes and short follow-up periods, and the long-term efficacy and safety of IS-targeted interventions therefore remain uncertain. Large, multicenter randomized controlled trials with sufficiently long follow-up are needed to determine whether reductions in IS levels translate into sustained renal and cardiovascular benefits. (ii) Standardization of IS assessment. Differences in analytical methods, sample handling, and reporting of IS concentrations may contribute to variability across studies. Establishing standardized and reproducible methods for IS measurement would improve the comparability of clinical and experimental findings and facilitate evidence synthesis. (iii) Feasibility and clinical translation of interventions. Sustained adherence to dietary modification remains challenging, while fecal microbiota transplantation (FMT), emerging dialysis technologies, and downstream pathway inhibitors require further evaluation of their safety, feasibility, and clinical applicability. Future research should therefore consider not only biochemical efficacy but also treatment adherence, safety, real-world feasibility, and long-term survival outcomes. (iv) Precision approaches and biomarker discovery. Interindividual differences in gut microbiota and tryptophan metabolism may influence IS production and treatment responses. Integrating microbiome sequencing with metabolomics, transcriptomics, and proteomics, together with artificial intelligence-assisted analyses, may help identify patient subgroups, clarify the microbiota–IS–host interaction network, and identify biomarkers associated with treatment response.

Overall, future progress will require rigorous clinical validation, standardized IS measurement, feasible intervention strategies, and better identification of patients who are most likely to benefit from specific IS-targeted approaches. These efforts may ultimately support more effective and personalized IS-targeted therapies in CKD.

## 6 Conclusion

IS is a gut-derived uremic toxin that has been implicated in CKD progression. Although substantial advances have been made in elucidating its pathogenic mechanisms and developing IS-targeted interventions, several challenges remain unresolved. Current therapeutic strategies, including dietary modulation, microbiota-based therapies, AST-120, modified dialysis techniques, and traditional Chinese medicine, are mainly supported by experimental studies and small-scale clinical trials, whereas robust evidence from large, long-term randomized controlled studies is still lacking. In addition, individual variations in IS metabolism, the absence of standardized monitoring approaches, and uncertainties regarding long-term efficacy and safety limit clinical translation. Future research should integrate multi-omics approaches, microbiome profiling, and precision medicine to better characterize individual differences in IS metabolism and optimize IS-targeted interventions, while the development of novel adsorption materials and combination strategies may provide additional therapeutic options. With continued mechanistic investigations and clinical validation, IS-targeted therapies may hold promise as a component of personalized CKD management, potentially contributing to improved long-term patient outcomes.
